# From hospital to community? The good, the bad, and the ugly of the 10-year plan to move care to the community

**DOI:** 10.3399/BJGP.2025.0567

**Published:** 2025-10-01

**Authors:** Luisa M Pettigrew, Jake Beech, Hugh Alderwick, Judith Smith

**Affiliations:** 1 The Health Foundation, London, UK; 2 London School of Hygiene & Tropical Medicine, London, UK; 3 Health Services Management Centre, University of Birmingham, Birmingham, UK

Labour’s 10-year health plan for England, published on 3 July, set out three strategic shifts — from hospital to community, sickness to prevention, and analogue to digital.^1^ This editorial focuses on the first — shifting care from hospital to community — and considers its implications for general practice, drawing on existing evidence to explore the good, the bad, and the potentially ugly.

The proposed shift from hospital to community hinges on the development of Neighbourhood Health Services, delivered by multidisciplinary community-based teams — including GPs, nurses, pharmacists, paramedics, and a mix of other staff. The plan also proposes Neighbourhood Health Centres (NHCs), to be open at least 12 hours a day, 6 days a week, and offering co-located NHS, local authority, and voluntary sector services. To incentivise the delivery of these services, the plan proposes two new neighbourhood contracts — a ‘single neighbourhood’ provider contract aiming to build on primary care networks (PCNs), covering a population of around 50 000; and a ‘multi-neighbourhood’ contract to cover a population of around 250 000, aiming to support back-office functions, digital transformation, estates, data analytics, and quality improvement. While large-scale GP organisations, such as federations, may be able to take on multi-neighbourhood contracts, they will also be open to NHS Trusts. Operating at a larger scale, integrated health organisations (IHOs) are also proposed. To be led by the ‘very best’ NHS Foundation Trusts, IHOs would hold the whole health budget for a defined population. The plan states these will become the ‘norm’ over time.

## The good

There are welcome aspects of the plan for primary care. First, the plan is ambitious, making a strong case for change. It draws attention to pressures facing the NHS and the need to shift care closer to home, which builds on efforts already underway to scale-up community-based services and aligns with long-standing aspirations to move away from hospital-centric models.^2–4^ Second, it provides a welcome focus on prevention and recognises the importance of the social determinants of health, proposing, for example, to co-locate employment and debt support services within NHCs. Third, the plan recognises long-standing inequities in health and care, and signals a review of the general practice funding formula to address disparities in resource allocation.

*“To be led by the ‘very best’ NHS Foundation Trusts, integrated health organisations would hold the whole health budget for a defined population.”*

In addition, NHS leaders have described an intention for a ‘tight-loose-tight’ approach: being tight on aims, loose on the mechanisms to achieve them, and tight on measuring outcomes.^5^ If this degree of local flexibility is realised, primary care teams and other local organisations will have some freedom to develop Neighbourhood Health Services in ways that best meet local needs while staying aligned with national goals.

## The bad

A big problem with the plan is that it lacks an implementation strategy and does not sufficiently engage with lessons from past initiatives. Integrated Care Pioneers and Vanguard New Care Models in the 2010s, which aimed to bring together primary, community, and secondary care providers under new contracts, failed to scale due to contracting complexity, funding uncertainty, and limited evidence of impact.^6–9^ In the late 2000s, Darzi Polyclinics were labelled ‘white elephants’ as they were deemed to be costly, often duplicative of other services, and viewed as a threat by many GPs.^10^ NHS walk-in centres received similar criticisms.^11^ NHCs, if not carefully planned and integrated with existing general practices, may face the same fate.

Financially, the plan rests on optimistic assumptions. The 2.8% annual funding uplift to the NHS announced in the spending review is below the long-term historical average of 3.7% and far below the major increases seen during the 2000s under the last Labour government (averaging 6.8%).^12^ In the absence of additional overall funding, the plan proposes a shift of resources away from the acute sector suggesting that £2.2 billion savings from reducing hospital deficits will be reinvested into primary care. Given that many hospitals have been in deficit for years and face major service pressures, it is unclear how such savings might be made and then moved to primary care.^13^

*“Some long-term benefits from the proposals in the 10-year plan are achievable, but only if they are underpinned by clear and realistic goals, meaningful frontline engagement, and sufficient upfront investment ...”*

Compounding these challenges is the fact that NHS England is being abolished and integrated care boards are undergoing 50% cost reductions, leading to inevitable mergers and the associated distraction of another ‘redisorganisation’.^14^ These organisations, however imperfect, have played critical roles in policy development, primary care support, and contract oversight; therefore raising serious questions about who will support and oversee the shift to community-based care.

The plan also draws on selective evidence. For example, it cites what seems to be a back-of-the-envelope calculation that time saved by GPs using automated voice technology will be equivalent to adding 2600 full-time equivalent GPs, and while it pledges to train 'thousands more GPs' it lacks a focus on how to stem the loss of GPs from NHS general practice. In addition, assumptions about the economic returns of integrated care are not supported by consistent evidence — while better integrated multidisciplinary care can improve patient satisfaction and access, the evidence on its impact on costs and outcomes is mixed.^6,7,15–18^

## The (potentially) ugly

The plan clearly opens the door to vertical integration where hospital NHS Trusts take on the running of general practice and other community services (or vice versa), as well as horizontal integration between general practice and community providers. Vertical integration policies seem to draw inspiration from accountable care organisations (ACOs) in the US.^9^ However, UK experience remains limited. In 2023, only around 1% of general practices in England were part of an NHS Trust, with the motivation for such arrangements often being to help stabilise practices facing potential closure, and only a handful operating at scale.^19–21^ Vertical integration can bring benefits such as economies of scale, better coordination between services, and more opportunities for career progression, but it can also lead to a loss of autonomy, responsiveness, and increased bureaucracy for primary care.^22^ Two studies in England have found modest reductions in hospital use and potential for savings from vertical integration; however, further research is needed to assess long-term outcomes, cost-effectiveness, and scalability.^16,17^

How the proposed new neighbourhood health contracts will work with the existing GP contract is currently unclear. For many GPs the idea of becoming part of an NHS Trust or a polyclinic-style model may feel threatening or unwelcome, and things may get ugly during contract negotiations. However, the context has changed since previous attempts at integrated care and polyclinics in the NHS. Practices have become accustomed to working at a larger scale and many function across multiple sites.^19^ PCNs have required practices to work together and diversify the workforce, with GPs now representing only 16% of the general practice full-time equivalent workforce and GP partners making up less than half of GPs.^23^ As a result, there may be less resistance to alternative contracts and NHCs. Moreover, at-scale GP organisations may see the plan as an opportunity to increase their reach. Notably, evidence from the US found that ACOs associated with greater savings include those led by independent physician groups and those with a larger proportion of primary care providers compared with those led by hospitals.^24^

## What next?

Some long-term benefits from the proposals in the 10-year plan are achievable, but only if they are underpinned by clear and realistic goals, meaningful frontline engagement, and sufficient upfront investment.^6^ Change at this scale requires time and goodwill from the workforce. Looking back, the NHS is at least a decade into efforts to scale-up general practice and integrate services. While the plan creates a sense of urgency, there must also be realism about the pace of reform. Given the limited and uncertain evidence base, iterative evaluation will be essential to understand what works and adapt accordingly. Ultimately, the greatest risk to delivery may not be operational, but political — with the most likely reason for derailment being another major reorganisation before 2035.
